# Integrating genetic and transcriptomic data to identify genes underlying obesity risk loci

**DOI:** 10.1038/s41366-025-01898-z

**Published:** 2025-09-26

**Authors:** Hanfei Xu, Shreyash Gupta, Ian Dinsmore, Abbey Kollu, Anne Marie Cawley, Mohammad Y. Anwar, Hung-Hsin Chen, Lauren E. Petty, Sudha Seshadri, Misa Graff, Jennifer E. Below, Jennifer A. Brody, Geetha Chittoor, Susan P. Fisher-Hoch, Nancy L. Heard-Costa, Daniel Levy, Honghuang Lin, Ruth J. F. Loos, Joseph B. Mccormick, Jerome I. Rotter, Tooraj Mirshahi, Christopher D. Still, Anita Destefano, L. Adrienne Cupples, Karen L. Mohlke, Kari E. North, Anne E. Justice, Ching-Ti Liu

**Affiliations:** 1 Department of Biostatistics, School of Public Health, Boston University, Boston, MA USA; 2 Department of Population Health Sciences, Geisinger, Danville, PA USA; 3 Department of Genomic Health, Geisinger, Danville, PA USA; 4 Department of Psychology and Neuroscience, University of North Carolina, Chapel Hill, NC USA; 5 Marsico Lung Institute, University of North Carolina, Chapel Hill, NC USA; 6 Department of Epidemiology, Gillings School of Global Public Health, University of North Carolina, Chapel Hill, NC USA; 7 Institute of Biomedical Sciences, Academia Sinica, Taipei, Nangang District Taiwan; 8 Vanderbilt Genetics Institute, Division of Genetic Medicine, Vanderbilt University Medical Center, Nashville, TN USA; 9 Department of Neurology, School of Medicine, Boston University, Boston, MA USA; 10 Glenn Biggs Institute for Alzheimer’s & Neurodegenerative Diseases, UT Health San Antonio, San Antonio, TX USA; 11 Department of Medicine, Cardiovascular Health Research Unit, University of Washington, Seattle, WA USA; 12 Department of Epidemiology, School of Public Health, UT Health Houston, Regional Academic Health Center, Brownsville, TX USA; 13 Framingham Heart Study, Framingham, MA USA; 14 Department of Neurology, Chobanian & Avedisian School of Medicine, Boston University, Boston, MA USA; 15 Population Sciences Branch, National Heart, Lung, and Blood Institute of the National Institutes of Health, Bethesda, MD USA; 16 Department of Medicine, University of Massachusetts Chan Medical School, Worcester, MA USA; 17 Charles Bronfman Institute for Personalized Medicine at Mount Sinai, Icahn School of Medicine at Mount Sinai, New York, NY USA; 18 Novo Nordisk Foundation Center for Basic Metabolic Research, Faculty of Health and Medical Sciences, University of Copenhagen, Copenhagen, Denmark; 19 Department of Pediatrics, The Institute for Translational Genomics and Population Sciences, The Lundquist Institute for Biomedical Innovation at Harbor-UCLA Medical Center, Torrance, CA USA; 20 Center for Obesity and Metabolic Health, Geisinger, Danville, PA USA; 21 Department of Genetics, School of Medicine, University of North Carolina, Chapel Hill, NC USA; 22 Department of Epidemiology, UTHealth Houston School of Public Health- Brownsville Campus, Brownsville, TX USA; 23 Department of Internal Medicine, McGovern Medical School, Brownsville, TX USA

**Keywords:** Obesity, Genetics

## Abstract

**Background:**

Genome-wide association studies (GWAS) have identified numerous body mass index (BMI) loci. However, most underlying mechanisms from risk locus to BMI remain unknown. Leveraging omics data through integrative analyses could provide more comprehensive views of biological pathways on BMI.

**Methods:**

We analyzed genotype and blood gene expression data from up to 5619 samples in the Framingham Heart Study (FHS). Using 3992 single-nucleotide polymorphisms (SNPs) at 97 BMI loci and 1408 transcripts within 1 Mb, we performed separate association analyses of transcript with BMI and SNP with transcript (*P*_BMI_ and *P*_SNP_, respectively) and then a correlated meta-analysis between the full summary data sets (*P*_META_). Transcripts were prioritized if we identified transcripts that met Bonferroni-corrected significance within each omic, showed stronger associations in the correlated meta-analysis than each omic, and had corresponding SNPs in the SNP-transcript-BMI association that were at least nominally associated with BMI in FHS data. We tested for generalization of identified association in a Hispanic ancestry sample of blood gene expression data and other samples in hypothalamus, nucleus accumbens, liver, and visceral adipose tissue (VAT) with significant threshold: *P*_META_ < 0.05 & *P*_META_ < *P*_SNP_ & *P*_META_ < *P*_BMI_.

**Results:**

Among 308 significant SNP-transcript-BMI associations, we identified seven genes (*NT5C2*, *GSTM3*, *SNAPC3*, *SPNS1*, *TMEM245*, *YPEL3*, and *ZNF646*) in five association regions. We generalized results for *SNAPC3* and *YPEL3* in Hispanic ancestry sample, for *YPEL3* in the nucleus accumbens, *ZNF646* and *GSTM3* in VAT, and *NT5C2*, *SNAPC3*, *TMEM245*, *YPEL3*, and *ZNF646* in liver.

**Conclusion:**

The identified genes help link the genetic variation at obesity-risk loci to biological mechanisms and health outcomes, thus translating GWAS findings to function.

## Introduction

Obesity is an enormous global public health burden. Since obesity is a major risk factor for numerous health outcomes, including cardiometabolic diseases [1], the rapid increase in the global obesity burden requires immediate public health action and a better understanding of obesity pathogenicity to prevent it. Decades of research, including genome-wide association studies (GWAS), have demonstrated the fundamental role of genetic susceptibility in obesity risk [2–6]. Each GWAS-identified locus potentially provides novel biologic insight; yet identifying the functional variants, genes, and underlying pathways at these loci has limited translation for precision medicine.

A major barrier to precision medicine for obesity has been the identification of functional genes underlying GWAS findings. Of the thousands of genomic regions associated with obesity-related traits by GWAS, over 90% are in non-coding, potentially regulatory regions of the genome [7]. Previous work mapping body mass index (BMI)-related genes implicates the involvement of synaptic function and glutamate receptor signaling, which impinge on key hypothalamic circuits that respond to changes in feeding and fasting and are regulated by key obesity-related molecules such as *BDNF* and *MC4R* [8, 9]. These pathways overlap with a proposed mechanism of action of topiramate, a component of one new FDA-approved weight-loss drug [10, 11]. However, our understanding of the fundamental mechanisms underlying genetic risk for obesity is limited and controversial even for *FTO*, with the most prominent effects on BMI [12].

Transcriptomics lie along pathways linking genetic susceptibility to obesity and is emerging as powerful disease biomarkers [13, 14] that may provide targetable “mechanistic bridges” linking GWAS findings with obesity risk. Large-scale characterization and integration of OMICs have been challenging because the comprehensive collection of molecular data has, until very recently, been either unavailable or cost-prohibitive in the context of a single study. However, OMICs scans in the same individuals in which obesity-associated loci discoveries were made are now available [15, 16], thereby facilitating comprehensive and efficient integration with genetic data to illuminate the underlying genes and mechanistic pathways of obesity-associated loci. Thus, studies that integrate GWAS with transcriptomics may lead to breakthroughs that reveal the genes contributing to obesity, identify individuals or groups that could benefit from aggressive prevention or treatment [17], or the repurposing of therapeutics [18].

Whole blood tissue is a key metabolic tissue critical to understanding obesity and further precision medicine. Blood is a sentinel tissue and a system integrator of tissue and organ-level perturbation in its physiological role; so all major metabolic perturbations may lead to adaptive responses in blood [19]. Additionally, blood is an easily accessible and minimally invasive tissue source clinically, making it an ideal starting point for research on molecular assessments of whole-blood OMICs contributing to the development of precision prevention, diagnosis, and treatment. Previous studies have shown that BMI-associated genes are highly enriched in brain tissue, including the hypothalamus and other regions of the brain involved in memory, appetite regulation, and metabolism [6]. Similarly, obesity-related genes involved in energy homeostasis (i.e. *SEC16B* [20], *HMGCR* [21], etc*)* are also expressed in the liver [22], a metabolically active organ [23], and human and mouse studies have shown associations between BMI genes and non-alcoholic fatty liver disease [24]. Therefore, our study used samples from whole blood tissue in the discovery stage, providing a strong foundation for generalizing to other potentially relevant tissues.

In this study, we analyzed GWAS data and transcriptomic data generated in whole blood in 5619 participants from the Framingham Heart Study (FHS) to identify potential causal genes through which known loci operate on obesity phenotypes (BMI). We used a correlated meta-analysis procedure to efficiently screen loci for potential candidate genes that are jointly associated with BMI and SNPs in linkage disequilibrium (LD) with established BMI-associated GWAS SNPs and follow-up promising associations in other obesity-relevant tissues.

## Methods

### Study sample

We included participants from both the Offspring cohort and the third generation (Gen3) cohort of the FHS. The Offspring cohort of FHS began in 1971 and consisted of children of the Original cohort and spouses of these children [25]. Gen3 cohort comprised children from the offspring families enrolled in 2002 [26]. The time intervals between clinical examinations for Offspring and Gen3 cohorts were approximately 4–6 years.

Since the timing of the blood sample taken for RNA collection was close to the eighth clinical examination (Exam 8) for the Offspring cohort and the second clinical examination (Exam 2) for the Gen3 cohort, our study was restricted to subjects with available blood sample, genotype data, and BMI information in either Exam 8 of the Offspring study or Exam 2 of the Gen3 study.

### Data description

FHS participants were genotyped using the Affymetrix GeneChip Human Mapping 500 K Array Set and another Affymetrix 50 K gene-centric array. The genotype imputation was performed using the Michigan Imputation Server with HRC reference panel release 1.1 April 2016 (HRC r1.1).

Fasting peripheral whole blood samples (2.5 ml) were collected from FHS participants at the eighth clinical examination (Exam 8) of the Offspring cohort and the second clinical examination (Exam 2) of the Gen3 cohort. The details of RNA collection and expression data cleaning have been previously described [27]. In our study, we used the expression data that have been adjusted using technical covariates and blood count [28, 29].

Height and weight were measured at Exam 8 of the Offspring cohort and Exam 2 of the Gen3 cohort. BMI was then calculated by weight (kg)/height(m)^2^.

### SNP-transcript association and transcript-BMI association

We analyzed 3992 SNPs that are in LD (*r*^2^ > 0.8) with 97 previously reported BMI variants from GIANT BMI GWAS paper [6] and the 1408 transcripts with a start position within 1 Mb of these variants. These 97 loci were originally selected as they have been well replicated in more recent GWAS studies [5, 30], have generalized across multiple global populations [30–32], are in well-imputed regions of the genomes, and many still lack strong candidate genes; thus, causal genes at these loci are also still under investigation.

We performed two kinds of association modeling. The first was a SNP-transcript association model, with the transcript as the outcome, and the SNP genotype as the predictor, adjusting for covariates including age at expression data collection, sex, and cohort identifier. We performed this first model for every SNP-transcript pair, using a linear mixed effects model to account for relatedness. The second model assessed the association between transcript and BMI, with expression of the transcript as the outcome, and BMI as the predictor, adjusting for age at expression data collection, sex, cohort identifier, and familial relatedness. We performed the second model for each transcript separately. In this manuscript, we will denote the *p* value of the SNP from the first model as *P*_SNP_ and the *p* value of BMI from the second model as *P*_BMI_.

### Correlated meta-analysis and causal inference test

We used the correlated meta-analysis model of Province and Borecki [33] to account for the potential dependence between the SNP-transcript and transcript-BMI associations, thus correcting for type I error, while still maintaining power for discovery by empirically estimating the null distribution from the test statistics This correlated meta-analysis model estimated the degree of correlation between SNP-transcript and transcript-BMI associations, and corrected for the inflation of type-I error that would be observed in a traditional meta-analysis (that assumes the two associations are statistically independent). Our model used a tetrachoric correlation, which was less sensitive to contamination from the alternative hypothesis than the Pearson correlation, thus preventing over-correction of the correlation.

In our analysis, for every SNP, we estimated the covariance matrix $\Sigma$ between two association results (${Z}_{\mathrm{SNP}}={\Phi }^{-1}({P}_{\mathrm{SNP}})$ and ${Z}_{\mathrm{BMI}}={\Phi }^{-1}({P}_{\mathrm{BMI}})$) using tetrachoric correlation, and then we calculated ${Z}_{\mathrm{meta}}={(Z}_{\mathrm{SNP}}+{Z}_{\mathrm{BMI}})\, \sim N(0,\mathrm{sum}(\Sigma ))$ and ${P}_{\mathrm{meta}}=1-\Phi ({Z}_{\mathrm{meta}})$ for each SNP-transcript pair.

After performing the correlated meta-analysis, we further screened the results to identify transcripts that met Bonferroni-corrected significance for each omic and were more significant in the correlated meta-analysis than in each omic. Thus, we included five criteria: *P*_meta_ < *P*_SNP_, *P*_meta_ < *P*_BMI_, *P*_SNP_ < (0.05/1408) = 3.6 × 10^−5^, *P*_BMI_ < 3.6 × 10^−5^, and the SNPs in the identified SNP-transcript pairs should have at least nominal association (*p* < 0.05) with BMI in FHS. The first two criteria ensured that both the SNP-transcript and transcript-BMI associations contributed to the meta-analysis. The third and fourth criteria guaranteed the Bonferroni-corrected significance of each association. The last criterion restricted the SNPs to those at least nominally associated with BMI in FHS data. The analysis code is available upon request.

We also performed causal inference test (CIT) analysis on the signals identified in the correlated meta-analysis. CIT is a hypothesis testing approach to identify potential mediators of the effects of genetic variants on traits of interest [34, 35]. Considering a transcript as a potential mediator of an effect of SNP on BMI, CIT could simultaneously test four conditions contributing to causal relationship evaluation among SNP, transcript, and BMI, and produce an omnibus test p-value. The omnibus null hypothesis is that the null hypothesis is true in at least one of the four components. We also performed CIT in the reverse direction, where we considered BMI as a potential mediator of an effect of SNP on a transcript. For each transcript we identified from the correlated meta-analysis, we analyzed the most significant SNP from its SNP-transcript associations in CIT analysis to avoid multicollinearity in the model.

### Biological interrogation and functional annotation

Regulatory variants are more likely to drive correlated signals of gene expression and SNP association than coding variants. To characterize candidate regulatory variants, we used chromatin marks and other epigenomic data that define regulatory elements or link regulatory elements to gene transcription start sites. We focused on data sets for liver, and component cell types, especially preadipocytes, adipocytes, and hepatocytes. We compared them to other tissues because tissue-restricted regulatory elements may be more likely to be relevant and functional. The resources we considered include accessible chromatin based on the assay for transposase-accessible chromatin (ATAC-seq) or DNase hypersensitivity from brain, blood, and liver; histone mark and transcription factor ChIP-seq and chromatin states from ENCODE [36] used for visual inspection and to assess variant overlap with potential candidate cis regulatory elements (cCREs). Additional resources used for variant annotation as described in Supplementary Note 1 include GeneCards [37], OMIM [38], and GTEx [39].

### Generalization: Cameron County Hispanic Cohort (CCHC)

The CCHC was established on the Texas-Mexico border in 2004 [40]. This randomly ascertained community cohort currently comprises over 5000 people and is approximately 60% female. All CCHC individuals were genotyped using the Illumina MEGAEX array [41].

RNA sequencing of CCHC participants was conducted using stored whole blood with sufficient quantity and quality. Sample collection and transcriptome profiling were described in detail previously [42]. We implemented a negative binomial model in DESeq2 [43] to identify BMI genes with covariate adjustment for sex, age, 10 PEER factors [44], and filtered results using default thresholds (*N* = 934).

We performed eQTL mapping using the GTEx v8 pipeline [39]. We identified eQTLs in cis (within 1 Mb) for each gene using FastQTL [45] with adjustment for sex, RNA-seq batch, 5 genetic principal components (PCs), and 10 PEER factors.

### Generalization on liver tissue and visceral adipose tissue (VAT): MyCode Bariatric Surgery Program (BSP)

The MyCode™Community Health Initiative (MyCode) study is a healthcare-based population study in central and northeastern Pennsylvania with ~2 million patients [46, 47]. We leveraged existing transcriptomic profiling in the Geisinger Health System’s (GHS) Bariatric Surgery Program (BSP) study (*N* = 2224) to generalize observed joint associations from whole blood in FHS to liver (*N* = 2224) and VAT (*N* = 657) tissues. Liver tissue sample collection and transcriptome profiling were described in detail previously [48, 49]. VAT samples were collected during the same procedure as described for the liver on a subset of subjects in the BSP, following similar storage procedures, as described previously [50]. Association analyses were performed using FastQTL [45], adjusting for sex, age, self-identified race/ethnicity, the first three genomic PCs to control for ancestry, and 60 PEER factors [44].

### Generalization on brain tissue

Analyses of hypothalamus (*N* = 131) and nucleus accumbens (*N* = 198) were conducted on samples from three cohorts: the Framingham Heart Study (FHS), the Religious Orders Study (ROS), and the Rush Memory and Aging Project (MAP). Details of RNA sequencing of hypothalamus and nucleus accumbens and the transcript-BMI association analysis were described previously [51]. In brief, total RNA were isolated using QIAzol Lysis Reagent (Qiagen, Valencia CA) and purified using miRNeasy MinElute Cleanup columns. Clipped large RNA sequencing reads shorter than 50 nucleotides were removed, and clipped small RNA sequencing reads shorter than 15 or longer than 23 were removed. For quality control, the nucleotide trimming tool sickle [52] v 1.33 was applied with Phred quality threshold of 20, removing low quality ends of reads and once again applying the lower bound read length filter. Differential expression testing of last measured BMI was performed using linear regression with LIMMA. We restricted our analysis to samples with RIN > 3 and BRAAK score ≤4. Covariates considered were sex, cohort, age at death, and sequencing batch. For the eQTL analysis, we used FastQTL and adjusted for covariates: 5 first genetic PCs, PEER factors according to the GTEx recommendations (15 PEER factors for hypothalamus and 30 PEER factors for nucleus accumbens), sex, age at death, cohort, and sequencing batch. We further performed meta-analysis using *p* values of SNP-transcript and transcript-BMI associations via Fisher’s method [53], producing a meta-analyzed *p* value.

## Results

### Sample characteristics

The characteristics of samples included in the discovery correlated meta-analysis, and generalization analyses are shown in Supplementary Table 1. The age distribution was similar for FHS whole blood, CCHC whole blood, and BSP liver analyses, with a mean ranging from 47 to 58, and the brain analyses had relatively older subjects with a mean age of 88. All study samples had a larger proportion of females compared to males. The BSP sample had a relatively higher BMI compared to other study samples. FHS and BSP are dominantly European ancestry, while CCHC was 100% Hispanic/Latino.

### Correlated Meta-analysis

**Fig. 1:**
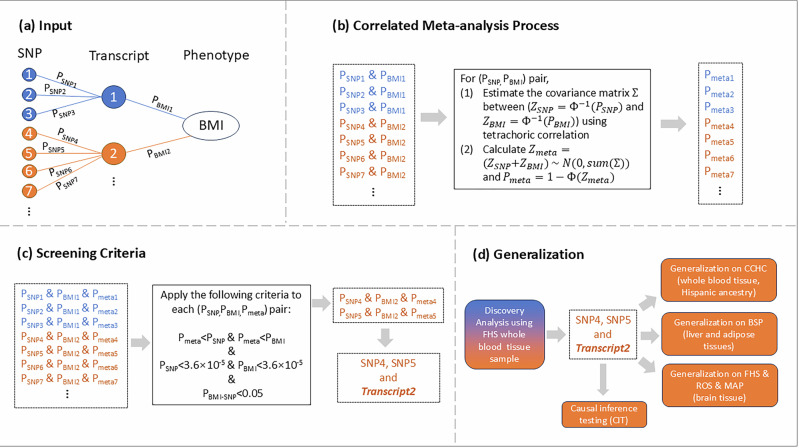
General workflow of the study design. **a** Step 1 included single omics associations for SNP to gene expression (*P*_SNP_) and gene expression to BMI (*P*_BMI_). **b** Step 2 included the correlated meta-analysis to account for the interdependence between *P*_SNP_ and *P*_BMI_. **c** Identifying all SNP—Gene—BMI combinations that met our filtering criteria, which included correlated meta-analysis results that are more significant than individual omics associations. **d** All significant SNP—Gene—BMI combinations were followed by generalization in blood, liver, adipose, brain tissues and causal inference testing (CIT) analysis.

Figure 1 shows the general workflow of our entire study. The models and filtering criteria of each step have been included in the “Methods” section. Separate results for suggestive SNP-transcript (*P*_SNP_ < (0.05/1408) = 3.6 × 10^−5^) and transcript-BMI (*P*_BMI_ < 3.6 × 10^−5^) signals in FHS analysis are provided in Supplementary Tables 2 and 3. The SNP-transcript analysis identified 3424 SNP-transcript pairs, including 1208 unique SNPs (corresponding to 31 GIANT BMI GWAS loci) and 74 unique transcripts. Genes, most frequently implicated with over 100 SNP-transcript analysis, were *AS3MT, DMXL2, NT5C2, PRKAG3, RQCD1, SNAPC3, TMOD2, TTLL4, USP37*, and *VIL1*. The most significant SNP-transcript pair was rs8049439-*TUFM* from 16p11.2 with *P*_SNP_ = 6.07 × 10^−203^. We did not observe inflation in the SNP-transcript analysis (Q-Q plot as Supplementary Fig. 1). The transcript-BMI analysis identified 306 transcripts with *P*_BMI_ < 3.6 × 10^−5^, corresponding to 78 GIANT BMI GWAS SNPs. The most significant transcript was *AHSP* at 16p11.2 with *P*_BMI_ = 1.60 × 10^−106^.

In the FHS correlated meta-analysis, we found 308 SNP-transcript-BMI associations corresponding to seven unique genes (*NT5C2*, *YPEL3*, *ZNF646*, *SPNS1*, *GSTM3*, *SNAPC3*, and *TMEM245*) potentially involved in transcriptional pathways from SNP to BMI (Table 1). 115 variants were involved in the SNP-transcript-BMI associations for *NT5C2*, including the reported BMI variant rs11191560. *YPEL3*, *ZNF646* and *SPNS1* were in the same region (16p11.2), and we observed 10, 46 and 91 SNP-transcript-BMI associations for *YPEL3*, *ZNF646* and *SPNS1* respectively, including three reported BMI variants rs4787491, rs9925964 and rs3888190. At the *TMEM245* locus, we pinpointed the SNP-transcript-BMI association to the reported BMI variant rs6477694. *GSTM3* was located at 1p13.3, with 4 SNP-transcript-BMI associations detected, including previously reported BMI signal rs17024393. *SNAPC3*, located at 9p22.3, had 41 SNP-transcript-BMI associations identified. In the directional causal analysis using CIT model, *YPEL3* reached nominal significance (*p* value = 0.011), indicating its role as a potential mediator between genetic variants and BMI.

**Table 1:** Summary table of significant genes and associations identified in discovery analysis and corresponding generalization results.

Gene symbol	GWAS SNP of locus	Number of SNPs interrogated in FHS	Number of significant SNP-transcript-BMI associations^a^					*p* value from CIT (SNP-transcript-BMI)^b^
			Discovery on FHS	Generalization on CCHC	Generalization on liver	Generalization on VAT	Generalization on brain—nucleus accumbens	
*NT5C2*	rs11191560	115	115		103			1.000
*GSTM3*	rs17024393	13	4			4		0.179
*SNAPC3*	rs4740619	225	41	37	40			1.000
*SPNS1*	rs3888190	91	91					1.000
*TMEM245*	rs6477694	1	1		1			1.000
*YPEL3*	rs4787491	10	10	10	10		10	0.011
*ZNF646*	rs9925964	46	46		15	46		0.188

Results for hypothalamus are removed, as no significant results were found.

^a^Filtering criteria for all generalization analyses: *P*_META_ < 0.05 & *P*_META_ < *P*_SNP_ & *P*_META_ < *P*_BMI_.

^b^The CIT analysis on the SNP-BMI-transcript direction showed no significant result.

### Generalization to Hispanic/Latino participants and other obesity-relevant tissues

**Fig. 2:**
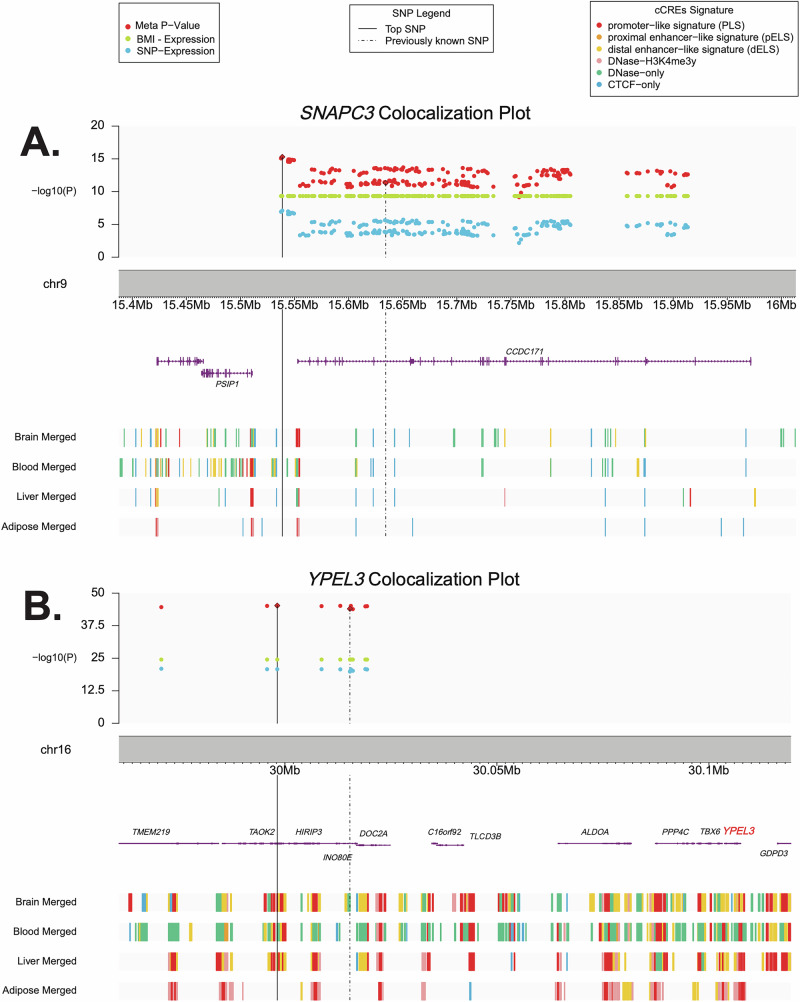
Regional association plot including association results for the discovery sample (Framingham Heart Study) for SNP with gene expression (blue), gene expression with BMI (green), and the correlated meta-analysis for SNP ~ gene expression ~ BMI (red). Annotation for potential candidate cis-regulatory elements from ENCODE are included for each reported SNP in the region. **A** *SNAPC3*, **B** *YPEL3*.

We tested for generalization of the above seven genes using CCHC blood gene expression data. Among the identified 308 SNP-transcript-BMI associations, 37 SNP-transcript-BMI associations corresponding to *SNAPC3* and 10 SNP-transcript-BMI associations corresponding to *YPEL3* remained significant (*P*_meta_< 0.05 & *P*_meta_ < *P*_SNP_ & *P*_meta_ < *P*_BMI_) (Supplementary Table 4). Regional association plots for each gene show annotation information (Fig. 2 and Supplementary Figs. 2–6). Of note, the top *P*_META_ SNP for *SNAPC3* and *YPEL3* are within or proximal to putative candidate cis-Regulatory Elements (cCREs) based on ENCODE [36] regulatory data on blood, brain, liver, and VAT tissues (Fig. 2).

**Fig. 3:**
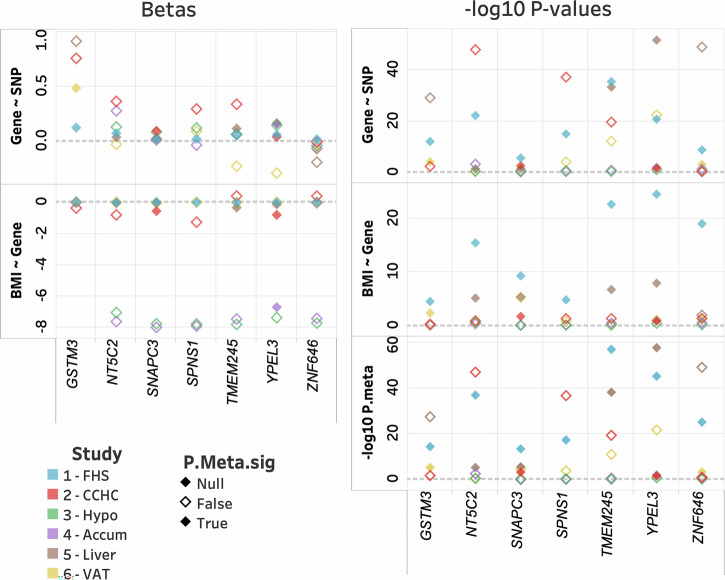
Summary of generalization for most significant SNP in discovery corelated meta-analysis. Results are provided for discovery sample (FHS, blue), generalization in blood (CCHC, red), and generalization to hypothalamus (Hypo, green), nucleus accumbens (Accum, purple), liver (brown) and VAT (yellow) tissues. We provide individual effect estimates and *p* values for each OMIC and meta-analysis. Filled diamonds indicate significant associations in the meta-analysis (Note: FHS is noted as NULL, as all are significant).

We also tested for generalization using gene expression in brain tissues. Hypothalamus tissue showed no significant SNP-transcript-BMI association. In contrast, the 10 SNP-transcript-BMI associations corresponding to *YPEL3* were significant in the generalization analysis on nucleus accumbens. Additionally, we were able to generalize signals in liver tissue for *NT5C2*, *SNAPC3*, *TMEM245*, *YPEL3*, and *ZNF646*, including 103, 40, 1, 10, and 15 SNP-transcript-BMI associations, respectively (Supplementary Table 4). Finally, we observed significant generalization in VAT for *GSTM3* and *ZNF646*, including 4 and 46 SNP-transcript-BMI associations, respectively. While the direction of effect was consistent for both brain tissues, even for non-significant associations (Table 2, Fig. 3 and Supplementary Table 4). The direction of effect was not always consistent across tissue types; however, consistency of direction of effect across various tissues may not be expected. Further work may be needed to clarify expectations of directional consistency across tissues with respect to BMI ~ Gene and SNP ~ associations.

**Table 2:** Summary results for most significant SNP–expression–BMI combination identified in Discovery (FHS) sample.

Gene	CHR	POS (b38)	dbSNP ID	EA	OA	GWAS tag SNP	GWAS tag dbSNP ID	Study	*P*_BMI_	*N*_BMI_	BETA_BMI_	*P*_SNP_	*N*_SNP_	BETA_SNP_	*P*_META_
*GSTM3*	chr1	109612066	rs17024393	C	T	Yes	rs17024393	FHS—Blood	2.67E−05	5619	0.003	9.30E−13	5257	0.123	**3.89E−15**
								CCHC—Blood	4.37E−01	934	−0.366	5.56E−03	769	0.757	1.70E−02
								Hypothalamus	9.48E−01	131	−7.858	–	–	–	–
								Nucleus accumbens	1.61E−01	198	−7.033	–	–	–	–
								Liver	7.06E−01	2225	0.007	6.56E−30		0.914	3.17E−28
								VAT	3.92E−03	657	−0.013	8.15E−05	657	0.484	**5.09E−06**
*NT5C2*	chr10	103154183	rs74233809	C	T	No	rs11191560	FHS—Blood	3.48E−16	5619	−0.004	5.82E−23	5257	0.072	**1.16E−37**
								CCHC—Blood	1.07E−01	934	−0.803	7.98E−49	769	0.363	9.76E−48
								Hypothalamus	2.02E−01	131	−7.037	4.30E−01	108	0.128	2.99E−01
								Nucleus accumbens	3.66E−01	198	−7.612	7.69E−04	170	0.276	2.59E−03
								Liver	6.40E−06	2225	−0.044	4.87E−02	2225	0.035	**4.98E−06**
								VAT	1.43E−01	657	0.007	5.90E−01	657	−0.031	2.94E−01
*SNAPC3*	chr9	15786904	rs10962158	A	T	No	rs4740619	FHS—Blood	4.71E−10	5619	0.002	2.97E−06	5257	0.015	**3.66E−14**
								CCHC—Blood	1.63E−02	934	−0.559	2.96E−03	769	0.091	**5.28E−04**
								Hypothalamus	6.41E−01	131	−7.751	2.88E−01	108	0.083	4.97E−01
								Nucleus accumbens	7.70E−01	198	−7.979	8.19E−01	170	0.011	9.21E−01
								Liver	4.03E−06	2225	−0.033	3.79E−02	2225	0.025	**2.55E−06**
								VAT	4.45E−06	657	−0.022	3.41E−01	657	0.019	2.18E−05
*SPNS1*	chr16	28814099	rs11860513	T	C	No	rs3888190	FHS—Blood	1.33E−05	5619	−0.001	1.03E−15	5257	0.019	**4.59E−18**
								CCHC—Blood	4.12E−02	934	−1.266	4.98E−38	769	0.293	1.85E−37
								Hypothalamus	6.62E−01	131	−7.764	2.38E−01	108	0.119	4.49E−01
								Nucleus accumbens	6.24E−01	198	−7.902	6.37E−01	170	−0.038	7.64E−01
								Liver	6.17E−01	2225	0.006	–	–	–	-
								VAT	1.13E−01	657	0.008	1.06E−04	657	0.088	1.48E−04
*TMEM245*	chr9	109170062	rs6477694	T	C	Yes	rs6477694	FHS—Blood	2.39E−23	5619	0.005	2.95E−36	5257	0.055	**1.09E−57**
								CCHC—Blood	3.91E−02	934	0.396	2.17E−20	769	0.337	4.20E−20
								Hypothalamus	7.22E−01	131	−7.796	5.13E−01	108	0.059	7.38E−01
								Nucleus accumbens	2.88E−01	198	−7.455	1.89E−01	170	0.062	2.13E−01
								Liver	1.72E−07	2225	−0.341	3.78E−34	2225	0.113	**6.08E−39**
								VAT	4.76E−01	657	−0.003	7.10E−13	657	−0.231	1.00E−11
*YPEL3*	chr16	29986879	rs4077410	G	A	No	rs4787491	FHS—Blood	3.19E−25	5619	0.007	1.68E−21	5257	0.057	**5.98E−46**
								CCHC—Blood	1.07E−01	934	−0.803	3.21E−02	769	0.037	**2.30E−02**
								Hypothalamus	3.22E−01	131	−7.365	1.07E−01	108	0.144	1.50E−01
								Nucleus accumbens	1.04E−01	198	−6.692	1.23E−02	170	0.156	**9.79E−03**
								Liver	1.09E−08	2225	−0.127	1.39E−52	2225	0.163	**2.10E−58**
								VAT	8.19E−02	657	0.008	3.89E−23	657	−0.294	1.76E−22
*ZNF646*	chr16	31077026	rs749671	A	G	No	rs9925964	FHS—Blood	9.68E−20	5619	−0.003	1.89E−09	5257	0.017	**7.33E−26**
								CCHC—Blood	3.91E−02	934	0.396	6.16E−01	769	−0.010	1.14E−01
								Hypothalamus	5.83E−01	131	−7.708	4.44E−01	108	−0.042	6.09E−01
								Nucleus accumbens	2.72E−01	198	−7.414	7.26E−02	170	−0.067	9.71E−02
								Liver	9.25E−03	2225	−0.020	7.32E−50	2225	−0.195	8.05E−50
								VAT	4.13E−02	657	0.010	9.75E−04	657	−0.081	4.48E−04

*EA* effect allele, *OA* other allele.

The bolded values in column “P_meta” means meeting significance criteria for generalization.

### Biological interrogation and functional annotation

Previous studies of gene function and bioinformatics characteristics (see Methods) of the significant genes highlight nearby signatures of gene regulation (Supplementary Note 1, Fig. 2 and Supplementary Figs. 2–6). Top SNPs from our correlated meta-analysis often coincide with regulatory elements, particularly in relevant tissues, while known index SNPs more often fell outside of these proposed regulatory elements. For example, our top SNP in *YPEL3* overlaps a likely regulatory element in three tissue types (brain, liver, and blood), but the previously known index variant overlaps a probable regulatory element in blood. Further, there is stronger evidence supporting the likely regulatory function by cCREs overlap for our lead SNP (Fig. 2B; i.e. promoter−like signature (PLS) for our lead SNP vs. DNase−only for known index SNP in blood). Similar evidence is observed for our top correlated SNP for *NT5C2* and *GSTM3*. For *ZNF646*, both the known and top correlated meta SNP are overlapping cCREs in multiple tissues. However, other loci do not provide evidence for cCREs overlap for either index SNP.

Seven genes were identified as significant in our correlated meta-analysis, many with potential relevance to metabolic and neurodevelopmental phenotypes (see Supplementary Note 1 for additional details). For example, *NT5C2*, a purine-metabolizing enzyme, is ubiquitously expressed and has been associated with reduced adiposity, obesity, and obesity-related depression [54–58]. Functional studies suggest its variants may influence BMI through miRNA-mediated regulation [59]. *YPEL3*, involved in glial development and apoptosis, has been linked to both BMI and schizophrenia, and mouse knockdown models showing altered fat composition, according to the International Mouse Phenotyping Consortium (IMPC) [60–62]. *GSTM3*, a detoxification enzyme, is highly polymorphic and associated with several cardiometabolic phenotypes, including hyperinsulinemia, type 2 diabetes, hypertension, and polycystic ovary syndrome (PCOS) [63–65]. Lastly, *SNAPC3*, a component of the small nuclear RNA transcription complex, is associated with schizophrenia and early-life growth trajectories via epigenetic regulation [66, 67]. These findings highlight the diverse biological pathways potentially contributing to metabolic and neuropsychiatric traits.

## Discussion

This study incorporated a correlated meta-analysis method to perform integrative analysis using genotype, gene expression, and phenotype (BMI) data. From the discovery analysis using the FHS whole blood data, we identified seven genes (*NT5C2*, *YPEL3*, *ZNF646*, *SPNS1*, *GSTM3*, *SNAPC3*, and *TMEM245*) that potentially lie along the pathway linking genetic variation to elevated BMI. Among those seven genes, *YPEL3* and *SNAPC3* associations were validated in whole blood in the CCHC study. In the analyses of tissues other than blood, *NT5C2*, *SNAPC3*, *TMEM245*, *YPEL3*, and *ZNF646* associations generalized in the liver tissue, *ZNF646* and *GSTM3* in VAT, and *YPEL3* in the nucleus accumbens.

Our literature search provides further details on potential roles for identified genes for obesity (Supplementary Note 1). *YPEL3* is located at 16p11.2, a gene dense region well-known for a microdeletion associated with neurocognitive developmental delay and predisposition to obesity [68–70]. Literature has reported that this region’s deletion event is related to a highly-penetrant form of obesity [71, 72], and is age- and gender-dependent [73, 74]. Within this region, *SH2B1* has received much attention as the likely causal gene underlying the mosaic effects of the 16p11.2 deletion and is thought to regulate body weight and glucose metabolism [75, 76]; and as a result, *YPEL3* has rarely been considered in previous studies. One of the previous studies that considered *YPEL3* [61] identified it as a pleiotropic gene jointly influencing BMI and risk of schizophrenia. In contrast, another study [77] asserted that the association between *YPEL3* and schizophrenia is due to its correlation with expression of *INO80E*, another possible candidate gene for BMI and risk of schizophrenia in the 16p11.2 region. Other model organism studies have shown alterations in *YPEL3* results in altered obesity phenotypes. For example, *YPEL3* knockdown in *Drosophila melanogaster* resulted in significant changes in body fat percentage [62]. Despite the controversial findings of *YPEL3* in the literature, several pieces of evidence support a role of *YPEL3* in BMI. First, the gene is highly expressed in whole blood and brain, similar to well-known BMI-related genes (Supplementary Note 1). Also, *YPEL3* was the sole candidate gene in this region identified by the current analysis and it showed significant causal SNP-transcript-BMI relationship in the CIT analysis. Further, the blood expression results were validated in an independent study of Hispanic participants, and the results generalized to both brain and liver tissues. Combined, this evidence suggests that more attention is warranted on this gene in the future.

*NT5C2* is located at 10q24.32, which has been reported as a highlight locus of autism spectrum disorder, brain arterial diameters, and schizophrenia [78–80]. *NT5C2* deletion was found to be protective in mice fed a high fat diet (HFD) [54]. A previous study in zebrafish found *NT5C2* as a potential causal gene in this region for blood pressure [81]. Notably, variation in this gene is also associated with lower visceral and subcutaneous fat [57], obesity, and the concurrence of obesity and depression [58] (Supplementary Note 1). Further, animal studies of *NT5C2* knock-outs show changes in body weight gain, insulin resistance on high-fat diet, and white adipose tissue mass [54, 56]. Kumar et al. found that rs11191548 decreased miRNA binding efficiency, which may explain the functional role of *NT5C2* influencing BMI [59]. Yet, our significant findings linking SNP variation to *NT5C2* gene expression with BMI in liver tissue is novel and a role for this gene in other tissues warrants further exploration. Literature shows strong support for *YPEL3* and *NT5C2* as likely candidate genes underlying the association with BMI in these two regions. However, existing knowledge that may offer a role for the other genes in the pathway to BMI is sparse.

While support for other genes identified herein is limited in the literature, *SNAPC3*, which validated in CCHC, and *TMEM245*, which generalized to liver tissue, have connections to obesity-related traits. For example, similar to both genes mentioned above, *SNAPC3* variants have also been associated with schizophrenia [66]. Also, DNA methylation in *SNAPC3* has been reported to mediate the association between breastfeeding and early-life growth trajectories [67]. The expression level of *TMEM245* has been associated with atrial fibrillation [82], and schizophrenia-associated variants have been reported within this gene [83].

In recent years, there has been growing interest in developing integrative approaches that utilize various OMICs data to uncover underlying biological mechanisms of obesity. Smemo et al. [12] found that obesity-associated variants within FTO were functionally connected with *IRX3 and IRX5* expression. Voisin et al. [84] and Tang et al. [85] evaluated the association and the interaction between obesity-associated SNPs and DNA methylation changes. Kogelman et al. [86] detected co-expression patterns among eQTLs, integrated with protein data, and detected several obesity candidate genes, such as *ENPP1*, *CTSL*, and *ABHD12B*. More recently, integrative analyses on multiple obesity and neuro-related phenotypes provided further gene lists that potentially affected relevant phenotypes jointly [61, 77]. Also, a recent study colocalized splice junction quantitative trait loci (sQTLs) measured in subcutaneous adipose tissue with 24 BMI GWAS loci, including with *YPEL3* [87], and another study has reported 162 BMI signals with a colocalized adipose eQTL [88].

When individual-level data is available, combining multiple OMICs datasets to perform further analysis is preferred [86, 89]. Yet, few integrative studies using summary-level data exist [61, 90], limiting cross study analyses. Thus, among all the integrative OMICs analyses, the correlation between OMICs is often ignored [90, 91]. In our study, we leveraged the correlated meta-analysis framework proposed [33], which is a robust approach to integrate “suspected” correlated SNP-transcript association and transcript-BMI association. This approach is useful for performing statistical integration and has been incorporated into many colocalization and polygenic pleiotropy detection methods [92, 93]. By performing correlated meta-analysis using summary level data, we ensured the correlation between summary statistics of OMICs scans were considered. Given the complex and potentially bidirectional relationship between gene expression levels and BMI, this approach is well-suited as it does not require an assumption about the direction of causation. Instead, this computationally simple, fast, and scalable approach can serve as a tool for refining or prioritizing known signals with diverse types of data, with the possibility of conducting follow-up analyses such as CIT to explore causal direction post-hoc. Indeed, we did identify evidence of potential causal relationships from SNP to BMI through expression of *YPEL3* in our CIT analyses. However, interpreting null findings at other genes is complex given the power requirements for causal inferences, relationships among genes for polygenic traits, the complex pathways that connect genetic variation to phenotypic expression, horizontal pleiotropy/epistasis, and limitations of the CIT to model bidirectional, direct and indirect effects. Thus, other mediating factors should be explored in future investigations.

There are potential limitations to our study. First, the data sets used in this analysis are limited in demographic variation. For example, there is a limited age range for brain-related tissues (mean age > 85 years for both tissues), as these tissues are available posthumously. Also, there is limited race/ethnic diversity in the discovery and liver and VAT generalization cohorts, all with primarily White/European Americans. These concerns are somewhat mitigated by the attempt to generalize associations from FHS to Hispanic/Latino participants of the CCHC. Another potential limitation is the differences in gene expression data collection between our cohort studies (i.e. array-based vs. RNAseq), which may limit the potential generalizations. Additionally, we used population-level cohort data for our analyses and thus expression data are measured under circumstances that may not be optimal for detecting relevant differences in gene expression^125^, and has the potential to influence both the strength and direction of gene expression. For example, SNP-associated gene expression changes in response to environmental exposures (i.e. high-fat foods, medications, etc). Last, we were unable to conduct in vitro or in vivo functional validation of the candidate genes and/or SNPs identified through our analyses.

Yet, compared to other integrative studies, our study has several strengths. To our knowledge, our study is the first one that takes the correlation between OMICs scans into the integrative analysis of BMI. We not only have a discovery study using whole blood samples from European ancestry, but validate these joint associations in an independent study of Hispanic participants, and generalize our findings to other tissues, including liver, VAT, and nucleus accumbens. Yet, our study has some limitations. First, the traditional meta-analysis instead of the correlated meta-analysis was used in the generalization analyses due to data sparsity. Also, we only included two types of OMICs data in our analyses, genetics and gene expression data. However, these analyses gave us a comprehensive view of how our findings can be interpreted across ancestry and tissue type. And, our work offers a framework for future investigations incorporating additional OMICs data, such as DNA methylation or protein data, as well as additional tissues, that can also be adopted for other traits of interest.

## Conclusion

Our study aimed to narrow in on causal genes that underly known obesity susceptibility loci. Specifically, we were interested in genetic variation that may be operating on variation in BMI through alterations in gene expression. Our integrative, multi-omics approach identified seven candidate genes within five genomic regions for BMI. Among these seven, we find the strongest support for *YPEL3*, *NT5C2*, and *SNAPC3*, through generalization across ethnicities, generalization across BMI-relevant tissues, and/or existing literature with a connection to BMI-related traits or gene functions. This deep dive into the etiology of obesity risk loci gets us one step forward to connecting genetic variation to biological mechanisms and health outcomes, and thus translating GWAS findings to function so that obesity precision treatment and prevention can begin.

## Supplementary information

Supplemental Note

Related Manuscript File

Supplemental Figures

## Data Availability

The individual level data are available on dbGAP (dbGAP ID: phs000007.v2.p1 and phs002611.v3.p16 for FHS and phs003894.v1.p1 for CCHC). MyCode data can be accessed by Geisinger investigators. There are restrictions to the sharing of MyCode DiscovEHR genetic datasets related to agreements between Geisinger and the Regeneron Genetics Center.
