# Supplementary material for: Integrating genetic and transcriptomic data to identify genes underlying obesity risk loci
Source: Int J Obes (Lond). 2025 Sep 26;49(11):2346–57. doi: 10.1038/s41366-025-01898-z (PMC12583137; doi:10.1038/s41366-025-01898-z)
Supplement: Supplementary file 1 — Supplemental Note [file 41366_2025_1898_MOESM1_ESM.docx]

**Supplementary Note 1.** Seven genes identified as significant in the discovery (FHS) analysis

***NT5C2. 5'-nucleotidase, cytosolic II*** is a protein-coding gene that may maintain internal composition of nucleotides. It hydrolyzes IMP (inosine monophosphate) and other purines (Genecards^1^). *Chr10:104,845,940-104,953,056* (GRCh37/hg19 by Ensembl^2^). No known monogenic conditions reported in OMIM (OMIM^3^). *NT5C2* is ubiquitously expressed (GTEx^4^) with the highest expression observed in the thyroid and esophagus. Mouse knockout models demonstrate reduced body weight gain, insulin resistance on high-fat diet, and white adipose tissue mass ^5,6^**. In-vitro studies in human skeletal muscle tissue show a** suppression of 5’-Nucleotidase enzymes that promote AMP-activated protein kinase (AMPK) phosphorylation and metabolism^7^, which may suggest metabolic flexibility in a condition of obesity. Genetic variations in *NT5C2* have been associated with lower visceral and subcutaneous fat^8^, obesity, and the concurrence of obesity and depression^9^. Two GWAS in East Asian populations identified *NT5C2,* rs113278154, as “associated with metabolically unhealthy phenotypes among normal weight individuals”^10,11^. Rs11191548 of *NT5C2*, which is in complete LD (All pop: R^2=.9815) with the GWAS index SNP in the region, rs11191560, was studied to determine if miRNAs in the region disrupted binding to their target gene in an allele specific manner^12^. The study found that rs11191548 altered luciferase activity and decreased miRNA binding efficiency, and thus could explain how *NT5C2* may be one of the functional genes influencing BMI.

***YPEL3. Yippee-Like 3*** is a protein-coding gene that is involved in the proliferation and apoptosis in myeloid precursor cells (Genecards). It is required for central and peripheral glial cell development, and mutation of *YPEL3* causes neuropathy^13^. *chr16:30,103,635-30,108,236* (GRCh37/hg19 by Ensembl). No known monogenic conditions reported in OMIM (OMIM). *YPEL3* is ubiquitously expressed (GTEx), with the highest expression observed in the whole blood and brain. Mouse knockout has a small body size and has neuronal irregularities, according to the International Mouse Phenotyping Consortium (IMPC). Liu and colleagues in 2020 identified *YPEL3* as a pleiotropic gene jointly influencing BMI and risk of schizophrenia, further supporting a neuronal correlation of this gene for obesity^14^. *YPEL3* knockdown in *Drosophila melanogaster* resulted in significant changes in body fat percentage^15^.

***ZNF646. Zinc Finger Protein 646*** is a protein-coding gene predicted to enable DNA-binding transcription factor activity, RNA polymerase II-specific, and RNA polymerase II cis-regulatory region sequence-specific DNA binding activity. *chr16:31,085,743-31,095,517* (GRCh37/hg19 by [Ensembl](https://grch37.ensembl.org/Homo_sapiens/Gene/Summary?g=ENSG00000167395)). *ZNF646* is ubiquitously expressed (GTEx) with the highest expression observed in the testis. No known monogenic conditions reported in OMIM (OMIM). Expression of *ZNF646* has been associated with Parkinson’s disease in two different studies^16,17^.

***TMEM245. Transmembrane protein 245*** is a protein-coding gene with no known function.

*chr9:111,777,432-111,882,225* (GRCh37/hg19 by Ensembl). *TMEM245* is ubiquitously expressed (GTEx) with the highest expression in the thyroid, ovaries, and adrenal glands. Zhang and colleagues reported an association between *TMEM245* gene expression levels and atrial fibrillation^109^. Variants in this gene have been associated with schizophrenia^18^, age of menarche^19^, body height^20^, and cognitive abilities^21^.

***SPNS1. Lysosomal H(+)-carbohydrate transporter*** is a protein-coding gene that functions in lysosomal recycling at a late stage of autophagy (Genecards). *SPNS1* also functions as a sphingolipid transporter and may be involved in necrotic or autophagic cell death (Genecards). *Chr16:28,985,542-28,995,869* (GRCh37/hg19 by Ensembl). No known monogenic conditions reported in OMIM (OMIM). *SPNS1* is ubiquitously expressed, with the strongest gene expression in arteries and the uterus. Differential expression of this gene has been associated with BMI^22^. This region has multiple coordinately regulated genes based on eQTL. In a recent in-silico study, *SPNS1* was differentially expressed in persons with T2D and obesity^23^. Polymorphisms in *SPNS1* have been associated with BMI^24^, asthma^24^, allergic disease^25^, and ADHD^26^ in GWAS.

***GSTM3.* Glutathione S-Transferase Mu 3** is a protein coding gene, an enzyme that belongs to the mu class and functions in the detoxification of electrophilic compounds, including carcinogens, therapeutic drugs, environmental toxins, and products of oxidative stress, by conjugation with glutathione. The genes encoding the mu class of enzymes are organized in a gene cluster on chromosome 1p13.3 and are known to be highly polymorphic. *GSTM3* may be involved in the uptake and detoxification of harmful compounds in the body at the testis and blood-brain barrier (Genecards). *chr1:110,276,554-110,284,384* (GRCh37/hg19 by Ensembl). No known monogenic conditions reported in OMIM (OMIM). *GSTM3* is ubiquitously expressed with the strongest expression in the testis and ovaries. *GSTM3* has been associated with hyperinsulinemia, T2D^27^, and hypertension^28^. Recent studies have reported an increase of *GSTM3* in the omental fat of polycystic ovary syndrome (PCOS)^29^.

***SNAPC3. Small nuclear RNA activating complex polypeptide 3 is*** part of the SNAPc complex required for the transcription of both RNA polymerase II and III small-nuclear RNA genes (Genecards). *SNAPC3* binds to the proximal sequence element (PSE), a non-TATA-box basal promoter element common to these 2 types of genes (GeneCard). *Chr9:15,422,702-15,465,951* (GRCh37/hg19 by Ensembl). *SNAPC3* is ubiquitously expressed with the strongest expression in the testis and the cerebellum. Variants in this gene have been associated with schizophrenia^30^. DNA methylation in *SNAPC3* mediates the association between breastfeeding and early-life growth trajectories^31^**.**

References:

1. Stelzer, G., Rosen, N., Plaschkes, I., Zimmerman, S., Twik, M., Fishilevich, S., Stein, T.I., Nudel, R., Lieder, I., Mazor, Y., et al. (2016). The GeneCards Suite: From Gene Data Mining to Disease Genome Sequence Analyses. Current Protocols in Bioinformatics *54*, 1 30 31-1 30 33. https://doi.org/10.1002/cpbi.5.

2. Yates, A.D., Achuthan, P., Akanni, W., Allen, J., Allen, J., Alvarez-Jarreta, J., Amode, M.R., Armean, I.M., Azov, A.G., Bennett, R., et al. (2020). Ensembl 2020. Nucleic Acids Research *48*, D682–D688. https://doi.org/10.1093/nar/gkz966.

3. Amberger, J.S., Bocchini, C.A., Scott, A.F., and Hamosh, A. (2019). OMIM.org: leveraging knowledge across phenotype-gene relationships. Nucleic Acids Research *47*, D1038–D1043. https://doi.org/10.1093/nar/gky1151.

4. Consortium, Gte. (2020). The GTEx Consortium atlas of genetic regulatory effects across human tissues. Science *369*, 1318–1330. https://doi.org/10.1126/science.aaz1776.

5. Johanns, M., Kviklyte, S., Chuang, S.J., Corbeels, K., Jacobs, R., Herinckx, G., Vertommen, D., Schakman, O., Duparc, T., Cani, P.D., et al. (2019). Genetic deletion of soluble 5’-nucleotidase II reduces body weight gain and insulin resistance induced by a high-fat diet. Molecular Genetics and Metabolism *126*, 377–387. https://doi.org/10.1016/j.ymgme.2019.01.017.

6. Camici, M., Garcia-Gil, M., Allegrini, S., Pesi, R., and Tozzi, M.G. (2020). Evidence for a Cross-Talk Between Cytosolic 5’-Nucleotidases and AMP-Activated Protein Kinase. Frontiers in Pharmacology *11*, 609849. https://doi.org/10.3389/fphar.2020.609849.

7. Kulkarni, S.S., Karlsson, H.K., Szekeres, F., Chibalin, A.V., Krook, A., and Zierath, J.R. (2011). Suppression of 5’-nucleotidase enzymes promotes AMP-activated protein kinase (AMPK) phosphorylation and metabolism in human and mouse skeletal muscle. Journal of Biological Chemistry *286*, 34567–34574. https://doi.org/10.1074/jbc.M111.268292.

8. Hotta, K., Kitamoto, A., Kitamoto, T., Mizusawa, S., Teranishi, H., Matsuo, T., Nakata, Y., Hyogo, H., Ochi, H., Nakamura, T., et al. (2012). Genetic variations in the CYP17A1 and NT5C2 genes are associated with a reduction in visceral and subcutaneous fat areas in Japanese women. Journal of Human Genetics *57*, 46–51. https://doi.org/10.1038/jhg.2011.127.

9. Samaan, Z., Lee, Y.K., Gerstein, H.C., Engert, J.C., Bosch, J., Mohan, V., Diaz, R., Yusuf, S., Anand, S.S., Meyre, D., et al. (2015). Obesity genes and risk of major depressive disorder in a multiethnic population: a cross-sectional study. Journal of Clinical Psychiatry *76*, e1611-1618. https://doi.org/10.4088/JCP.14m09720.

10. Park, J.M., Park, D.H., Song, Y., Kim, J.O., Choi, J.E., Kwon, Y.J., Kim, S.J., Lee, J.W., and Hong, K.W. (2021). Understanding the genetic architecture of the metabolically unhealthy normal weight and metabolically healthy obese phenotypes in a Korean population. Scientific Reports *11*, 2279. https://doi.org/10.1038/s41598-021-81940-y.

11. Wen, W., Zheng, W., Okada, Y., Takeuchi, F., Tabara, Y., Hwang, J.Y., Dorajoo, R., Li, H., Tsai, F.J., Yang, X., et al. (2014). Meta-analysis of genome-wide association studies in East Asian-ancestry populations identifies four new loci for body mass index. Human Molecular Genetics *23*, 5492–5504. https://doi.org/10.1093/hmg/ddu248.

12. Kumar, P., Traurig, M., and Baier, L.J. (2020). Identification and functional validation of genetic variants in potential miRNA target sites of established BMI genes. International Journal of Obesity (London) *44*, 1191–1195. https://doi.org/10.1038/s41366-019-0488-8.

13. Blanco-Sánchez, B., Clément, A., Stednitz, S.J., Kyle, J., Peirce, J.L., McFadden, M., Wegner, J., Phillips, J.B., Macnamara, E., Huang, Y., et al. (2020). yippee like 3 (ypel3) is a novel gene required for myelinating and perineurial glia development. PLoS Genetics *16*, e1008841. https://doi.org/10.1371/journal.pgen.1008841.

14. Liu, H., Sun, Y., Zhang, X., Li, S., Hu, D., Xiao, L., Chen, Y., He, L., and Wang, D.W. (2020). Integrated Analysis of Summary Statistics to Identify Pleiotropic Genes and Pathways for the Comorbidity of Schizophrenia and Cardiometabolic Disease. Frontiers in Psychiatry *11*, 256. https://doi.org/10.3389/fpsyt.2020.00256.

15. Baranski, T.J., Kraja, A.T., Fink, J.L., Feitosa, M., Lenzini, P.A., Borecki, I.B., Liu, C.-T., Cupples, L.A., North, K.E., and Province, M.A. (2018). A high throughput, functional screen of human Body Mass Index GWAS loci using tissue-specific RNAi Drosophila melanogaster crosses. PLoS Genet *14*, e1007222. https://doi.org/10.1371/journal.pgen.1007222.

16. Li, C.Y., Ou, R.W., Chen, Y.P., Gu, X.J., Wei, Q.Q., Cao, B., Zhang, L.Y., Hou, Y.B., Liu, K.C., Chen, X.P., et al. (2021). Genetic Analysis of ZNF Protein Family Members for Early-Onset Parkinson’s Disease in Chinese Population. Molecular Neurobiology *58*, 3435–3442. https://doi.org/10.1007/s12035-021-02354-5.

17. Langmyhr, M., Henriksen, S.P., Cappelletti, C., van de Berg, W.D.J., Pihlstrøm, L., and Toft, M. (2021). Allele-specific expression of Parkinson’s disease susceptibility genes in human brain. Scientific Reports *11*, 504. https://doi.org/10.1038/s41598-020-79990-9.

18. Xu, C., Aragam, N., Li, X., Villla, E.C., Wang, L., Briones, D., Petty, L., Posada, Y., Arana, T.B., Cruz, G., et al. (2013). BCL9 and C9orf5 are associated with negative symptoms in schizophrenia: meta-analysis of two genome-wide association studies. PLoS One *8*, e51674. https://doi.org/10.1371/journal.pone.0051674.

19. Perry, J.R., Day, F., Elks, C.E., Sulem, P., Thompson, D.J., Ferreira, T., He, C., Chasman, D.I., Esko, T., Thorleifsson, G., et al. (2014). Parent-of-origin-specific allelic associations among 106 genomic loci for age at menarche. Nature *514*, 92–97. https://doi.org/10.1038/nature13545.

20. Sakaue, S., Kanai, M., Tanigawa, Y., Karjalainen, J., Kurki, M., Koshiba, S., Narita, A., Konuma, T., Yamamoto, K., Akiyama, M., et al. (2021). A cross-population atlas of genetic associations for 220 human phenotypes. Nature Genetics *53*, 1415–1424. https://doi.org/10.1038/s41588-021-00931-x.

21. Lee, J.J., Wedow, R., Okbay, A., Kong, E., Maghzian, O., Zacher, M., Nguyen-Viet, T.A., Bowers, P., Sidorenko, J., Karlsson Linnér, R., et al. (2018). Gene discovery and polygenic prediction from a genome-wide association study of educational attainment in 1.1 million individuals. Nature Genetics *50*, 1112–1121. https://doi.org/10.1038/s41588-018-0147-3.

22. Huan, T., Liu, C., Joehanes, R., Zhang, X., Chen, B.H., Johnson, A.D., Yao, C., Courchesne, P., O’Donnell, C.J., Munson, P.J., et al. (2015). A systematic heritability analysis of the human whole blood transcriptome. Human Genetics *134*, 343–358. https://doi.org/10.1007/s00439-014-1524-3.

23. Eldakhakhny, B.M., Al Sadoun, H., Choudhry, H., and Mobashir, M. (2021). In-Silico Study of Immune System Associated Genes in Case of Type-2 Diabetes With Insulin Action and Resistance, and/or Obesity. Frontiers in Endocrinology (Lausanne) *12*, 641888. https://doi.org/10.3389/fendo.2021.641888.

24. Zhu, Z., Guo, Y., Shi, H., Liu, C.-L., Panganiban, R.A., Chung, W., O’Connor, L.J., Himes, B.E., Gazal, S., Hasegawa, K., et al. (2020). Shared Genetic and Experimental Links between Obesity-Related Traits and Asthma Subtypes in UK Biobank. J Allergy Clin Immunol *145*, 537–549. https://doi.org/10.1016/j.jaci.2019.09.035.

25. Ferreira, M.A.R., Vonk, J.M., Baurecht, H., Marenholz, I., Tian, C., Hoffman, J.D., Helmer, Q., Tillander, A., Ullemar, V., Lu, Y., et al. (2019). Eleven loci with new reproducible genetic associations with allergic disease risk. Journal of Allergy and Clinical Immunology *143*, 691–699. https://doi.org/10.1016/j.jaci.2018.03.012.

26. Rao, S., Baranova, A., Yao, Y., Wang, J., and Zhang, F. (2022). Genetic Relationships between Attention-Deficit/Hyperactivity Disorder, Autism Spectrum Disorder, and Intelligence. Neuropsychobiology *81*, 484–496. https://doi.org/10.1159/000525411.

27. Pass, G.J., Becker, W., Kluge, R., Linnartz, K., Plum, L., Giesen, K., and Joost, H.G. (2002). Effect of hyperinsulinemia and type 2 diabetes-like hyperglycemia on expression of hepatic cytochrome p450 and glutathione s-transferase isoforms in a New Zealand obese-derived mouse backcross population. Journal of Pharmacology and Experimental Therapeutics *302*, 442–450. https://doi.org/10.1124/jpet.102.033553.

28. Delles, C., Padmanabhan, S., Lee, W.K., Miller, W.H., McBride, M.W., McClure, J.D., Brain, N.J., Wallace, C., Marçano, A.C., Schmieder, R.E., et al. (2008). Glutathione S-transferase variants and hypertension. Journal of Hypertension *26*, 1343–1352. https://doi.org/10.1097/HJH.0b013e3282fe1d67.

29. Cortón, M., Botella-Carretero, J.I., López, J.A., Camafeita, E., San Millán, J.L., Escobar-Morreale, H.F., and Peral, B. (2008). Proteomic analysis of human omental adipose tissue in the polycystic ovary syndrome using two-dimensional difference gel electrophoresis and mass spectrometry. Human Reproduction *23*, 651–661. https://doi.org/10.1093/humrep/dem380.

30. Goes, F.S., McGrath, J., Avramopoulos, D., Wolyniec, P., Pirooznia, M., Ruczinski, I., Nestadt, G., Kenny, E.E., Vacic, V., Peters, I., et al. (2015). Genome-wide association study of schizophrenia in Ashkenazi Jews. American Journal of Medical Genetics B Neuropsychiatric Genetics *168*, 649–659. https://doi.org/10.1002/ajmg.b.32349.

31. Briollais, L., Rustand, D., Allard, C., Wu, Y., Xu, J., Rajan, S.G., Hivert, M.F., Doyon, M., Bouchard, L., McGowan, P.O., et al. (2021). DNA methylation mediates the association between breastfeeding and early-life growth trajectories. Clinical Epigenetics *13*, 231. https://doi.org/10.1186/s13148-021-01209-z.
