# Supplementary material for: Integrating genetic and transcriptomic data to identify genes underlying obesity risk loci
Source: Int J Obes (Lond). 2025 Sep 26;49(11):2346–57. doi: 10.1038/s41366-025-01898-z (PMC12583137; doi:10.1038/s41366-025-01898-z)

Supplementary Figure 1. The Q-Q plot of p-values from SNP-transcript association analysis using Discovery (FHS) sample.

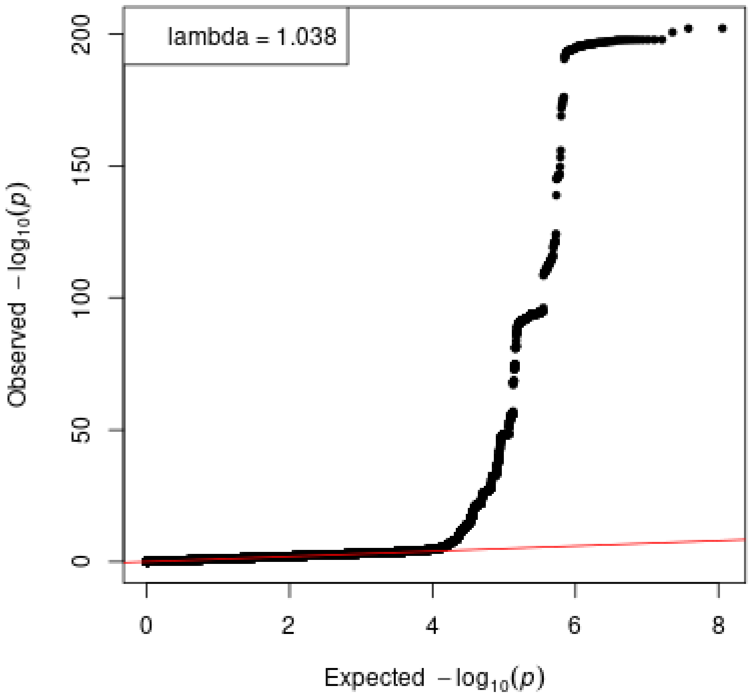

Supplementary Figure 2. Regional association plot for the *NT5C2* gene including association results for the discovery sample (Framingham Heart Study) for each tested SNP with gene expression (blue), gene expression with BMI (green), and the correlated meta-analysis for SNP ~ gene expression ~BMI (red). Annotation for potential ccREs from ENCODE are included for the region.

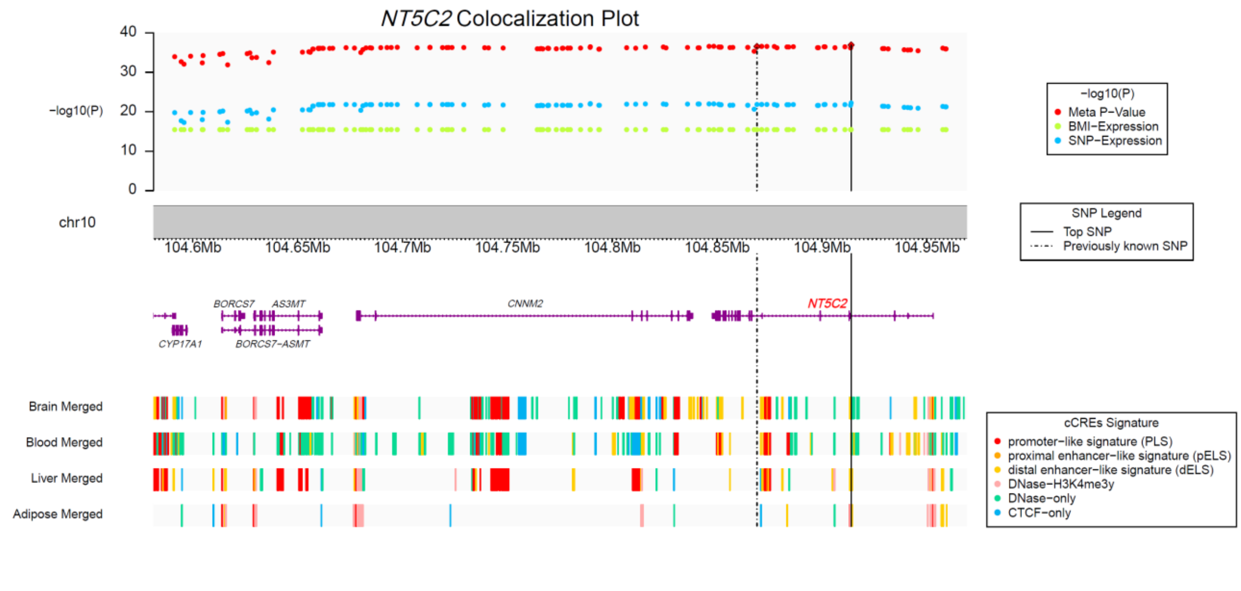

Supplementary Figure 3. Regional association plot for the *GSTM3* gene including association results for the discovery sample (Framingham Heart Study) for each tested SNP with gene expression (blue), gene expression with BMI (green), and the correlated meta-analysis for SNP ~ gene expression ~ BMI (red). Annotation for potential ccREs from ENCODE are included for the region.

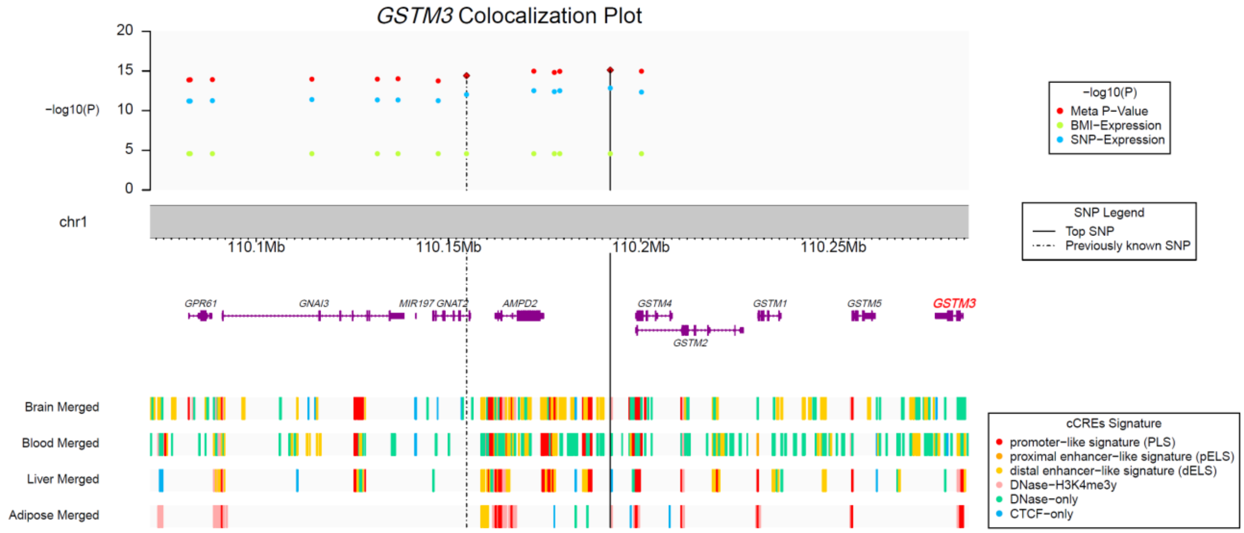

Supplementary Figure 4. Regional association plot for the *SPNS1* gene including association results for the discovery sample (Framingham Heart Study) for each tested SNP with gene expression (blue), gene expression with BMI (green), and the correlated meta-analysis for SNP ~ gene expression ~ BMI (red). Annotation for potential ccREs from ENCODE are included for the region.

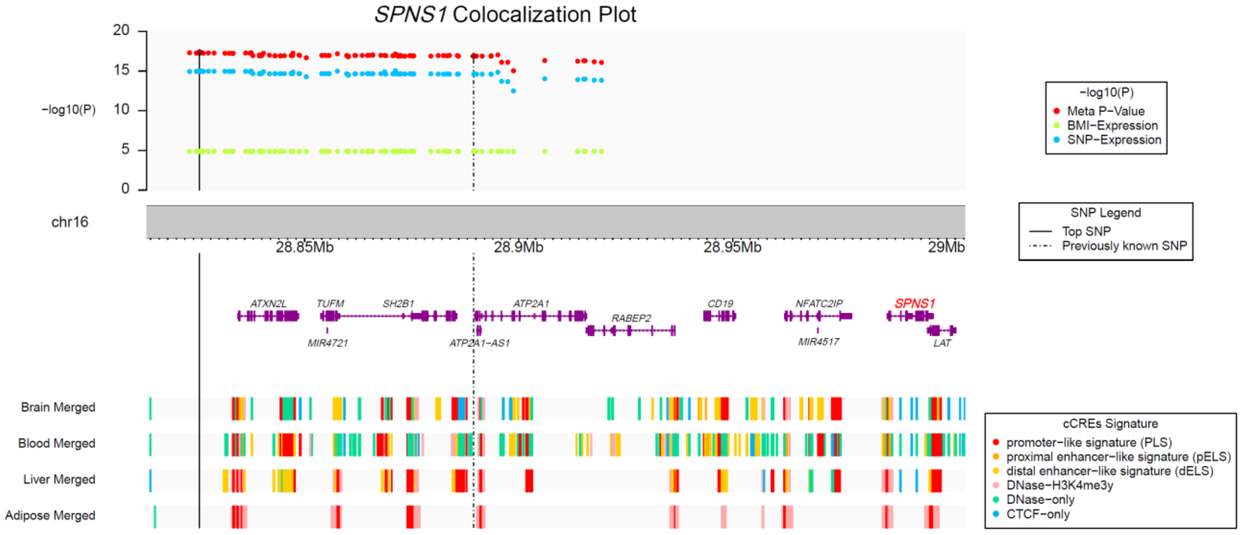

Supplementary Figure 5. Regional association plot for the *TMEM245* gene including association results for the discovery sample (Framingham Heart Study) for each tested SNP with gene expression (blue), gene expression with BMI (green), and the correlated meta-analysis for SNP ~ gene expression ~ BMI (red). Annotation for potential ccREs from ENCODE are included for the region.

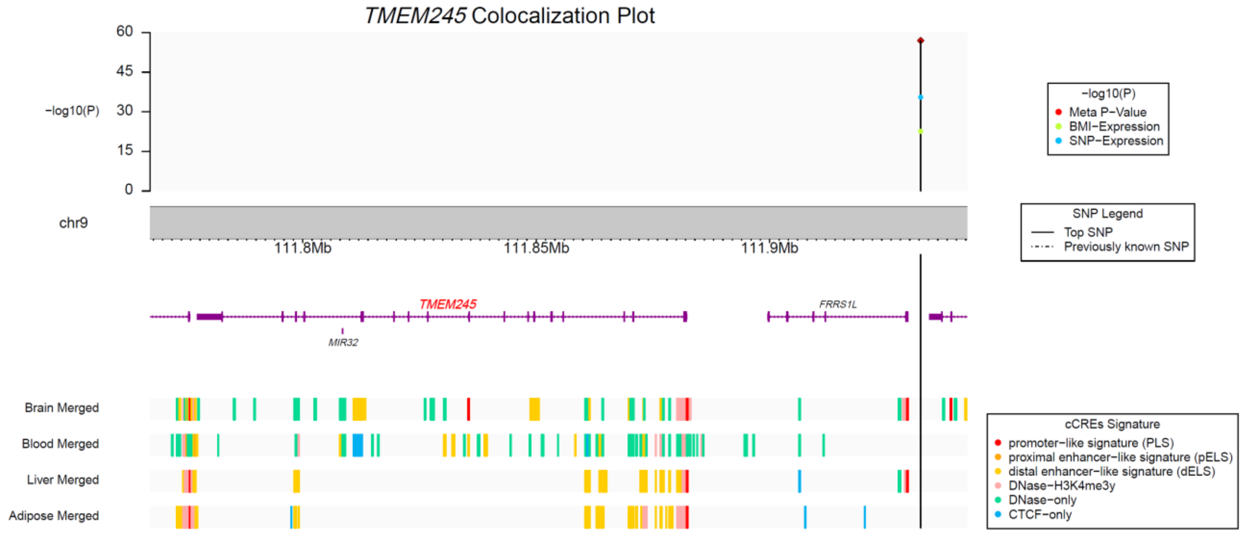

Supplementary Figure 6. Regional association plot for the *ZNF646* gene including association results for the discovery sample (Framingham Heart Study) for each tested SNP with gene expression (blue), gene expression with BMI (green), and the correlated meta-analysis for SNP ~ gene expression ~ BMI (red). Annotation for potential ccREs from ENCODE are included for the region.

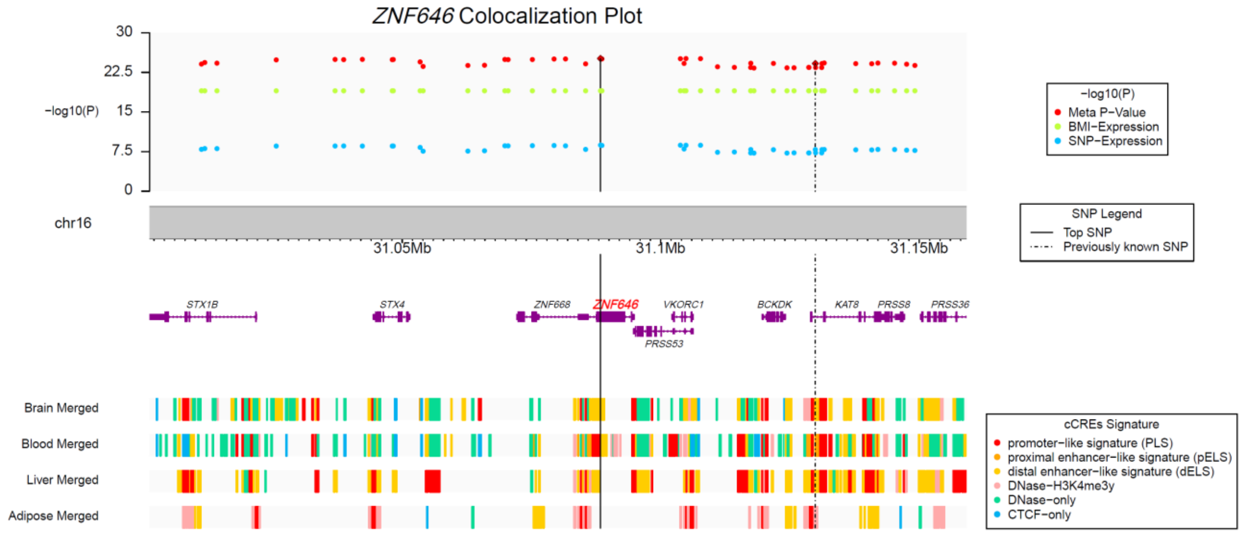

Supplement: Supplementary file 3 — Supplemental Figures [file 41366_2025_1898_MOESM3_ESM.pdf]
